# Neural crest-specific loss of *Bmp7* leads to midfacial hypoplasia, nasal airway obstruction and disordered breathing, modeling obstructive sleep apnea

**DOI:** 10.1242/dmm.047738

**Published:** 2021-02-11

**Authors:** Pranidhi Baddam, Vivian Biancardi, Daniela M. Roth, Farah Eaton, Claudine Thereza-Bussolaro, Rupasri Mandal, David S. Wishart, Amy Barr, Joanna MacLean, Carlos Flores-Mir, Silvia Pagliardini, Daniel Graf

**Affiliations:** 1 School of Dentistry, Faculty of Medicine and Dentistry, University of Alberta, Edmonton, AB T6G 2E1, Canada; 2 Department of Physiology, Faculty of Medicine and Dentistry, University of Alberta, Edmonton, AB T6G 2E1, Canada; 3 Department of Dentistry, Hospital dos Pinheiros, UNIFASIPE, Sinop, Mato Grosso 78550-000, Brazil; 4 The Metabolomics Innovation Centre, Department of Biological Sciences, Faculty of Science, University of Alberta, Edmonton, AB T6G 1C9, Canada; 5 Department of Pharmacology, Faculty of Medicine and Dentistry, University of Alberta, Edmonton, AB T6G 2E1, Canada; 6 Department of Pediatrics and the Women & Children's Health Research Institute, Faculty of Medicine and Dentistry, University of Alberta Edmonton, AB T6G 2E1, Canada; 7 Stollery Children's Hospital, Edmonton, AB T6G 2B7, Canada; 8 Department of Medical Genetics, Faculty of Medicine and Dentistry, University of Alberta, Edmonton, AB T6G 2H7, Canada

**Keywords:** Bone morphogenetic protein 7, Midfacial hypoplasia, Airway obstruction, Nasal septum deviation, Turbinate hypertrophy, Apneas, Sleep-related breathing disorders

## Abstract

Pediatric obstructive sleep apnea (OSA), a relatively common sleep-related breathing disorder affecting ∼1-5% of children, is often caused by anatomical obstruction and/or collapse of the nasal and/or pharyngeal airways. The resulting sleep disruption and intermittent hypoxia lead to various systemic morbidities. Predicting the development of OSA from craniofacial features alone is currently not possible, and controversy remains as to whether upper-airway obstruction facilitates reduced midfacial growth or vice versa. Currently, there is no rodent model that recapitulates both the development of craniofacial abnormalities and upper-airway obstruction to address these questions. Here, we describe that mice with a neural crest-specific deletion of *Bmp7* (Bmp7^ncko^) present with a shorter, more acute-angled cranial base, midfacial hypoplasia, nasal septum deviation, turbinate swelling and branching defects, and nasal airway obstruction. Interestingly, several of these craniofacial features develop after birth during periods of rapid midfacial growth and precede the development of an upper-airway obstruction. We identified that, in this rodent model, no single feature appeared to predict upper-airway obstruction, but the sum of those features resulted in reduced breathing frequency, apneas and overall reduced oxygen consumption. Metabolomics analysis of serum from peripheral blood identified increased levels of hydroxyproline, a metabolite upregulated under hypoxic conditions. As this model recapitulates many features observed in OSA, it offers unique opportunities for studying how upper-airway obstruction affects breathing physiology and leads to systemic morbidities.

This article has an associated First Person interview with the first author of the paper.

## Abstract (precis)

**Summary:** Using morphometric, respiratory function and metabolomics analyses, we demonstrate that the *Bmp7* neural crest knockout mouse is a model for nasal airway obstruction with a craniofacial origin, allowing the study of disordered breathing.

## INTRODUCTION

Sleep-related breathing disorders (SRBDs) encompass a spectrum of problems including obstructive sleep apnea (OSA), central sleep apnea and complex sleep apnea (Gipson et al., 2019; Morgenthaler et al., 2006). In children, pediatric OSA is the most common and widely investigated form of apnea, with a prevalence of 1.2-5.7% (Gipson et al., 2019). It is often caused by physical obstruction of either nasal and/or pharyngeal airways, leading to reduced airflow intake and/or intermittent apneas, resulting in difficulty to breathe. This can lead to intermittent hypoxia (IH) conditions most prominently noticed during sleep, and has been associated with a variety of systemic (including cardiovascular, metabolic and neurocognitive) morbidities, as well as behavioral problems at home and school that may continue and progress into adulthood (Gozal, 1998; Leger et al., 2012).

The etiology of pediatric OSA is multifactorial. Craniofacial and neuromuscular factors along with lymphoid tissue hypertrophy and airway soft tissue inflammation are considered critical components contributing to pediatric OSA (Bozzini and Di Francesco, 2016); however, controversy remains regarding the causes or consequences of these various manifestations. The resulting IH is considered a critical factor in the systemic pathogenesis of OSA (Dewan et al., 2015). The gold standard for diagnosis of OSA should include nocturnal polysomnography. Chronic untreated OSA can result in cardiovascular, neurocognitive and metabolic morbidities (Gabryelska et al., 2018), whereby the presence of IH is associated with increased severity of these morbidities (Veasey, 2009). Current surgical approaches are primarily aimed at relieving the physical obstruction (Hoeve et al., 1999). Hypertrophy of tonsils and adenoids can contribute to pharyngeal obstruction. Hence, a common pediatric surgical approach is tonsillectomy and adenoidectomy to remove enlarged lymphoid tissue (Carithers et al., 1987). However, upper-airway obstruction commonly remains as a post-operative symptom, residual pediatric OSA, suggestive of a more complex, multifactorial etiology of pediatric OSA/SRBD. The lack of complete resolution with these interventions also highlights the need to consider various craniofacial components in addition to adenoids (Carithers et al., 1987) for better screening, diagnosis and management outcomes.

Craniofacial abnormalities strongly predispose to upper-airway obstruction, as the prevalence of OSA increases to up to 67% in children with craniofacial syndromes (Skotko et al., 2017; Tan et al., 2016). Midfacial hypoplasia, nasal septum deviation, micrognathia, craniosynostosis, achondroplasia and cleft palate are all considered contributing factors to upper-airway obstruction (Cielo et al., 2016). A common denominator in all these abnormalities is altered or lack of craniofacial growth. However, it remains unclear whether altered growth predisposes to physical upper-airway obstruction or vice versa, because most clinical studies are static retrospective rather than long-term prospective (Cielo et al., 2016).

Various animal models have been used to assess the short- and long-term consequences of SRBD, ranging from experimentally induced hypoxia through the use of hypoxia chambers, forced sleep deprivation or naturally occurring obstruction models like that of the English bulldog (Hendricks et al., 1987). Risk factors for SRBD, such as obesity, have also been examined (Brennick et al., 2009; Polotsky et al., 2012; Strohl and Thomas, 2001). Whole-body plethysmography was used to assess changes to respiration in rodent models (Chopra et al., 2016). However, none of those models was able to clearly delineate the causes and consequences of pediatric OSA. As a result, it remains uncertain whether craniofacial abnormalities such as midfacial hypoplasia precede nasal airway obstruction or whether airway obstruction predisposes or enhances craniofacial abnormalities.

Pierre Robin sequence is frequently associated with craniofacial abnormalities such as micrognathia, midfacial hypoplasia and airway obstruction (Gangopadhyay et al., 2012). Deletion of bone morphogenetic protein 7 (*Bmp7*) in mice recapitulates many of those craniofacial features (Kouskoura et al., 2016). However, complete loss of *Bmp7* results in cleft palate and perinatal lethality (Kouskoura et al., 2013), precluding postnatal assessment of craniofacial growth. Mice with neural crest-specific deletion of *Bmp7* (Bmp7^ncko^) survive postnatally and present with micrognathia and midfacial hypoplasia (Kouskoura et al., 2016).

Using micro-computed tomography (µCT) and morphometrics, we describe here the sequence of when these midfacial abnormalities develop in Bmp7^ncko^ mice. Unrestrained whole-body plethysmography was used to characterize respiratory function under normoxic, hypoxic and hyperoxic conditions. Oxygen consumption and respiratory gas exchange were monitored using a Comprehensive Lab Monitoring System (CLAMS). An unbiased metabolomics analysis on blood serum was conducted to identify changes to metabolites at the onset of upper-airway obstruction. We demonstrate that a combination of craniofacial defects leads to nasal airway obstruction, and that craniofacial growth deficiencies precede nasal airway obstruction.

To our knowledge, this is the first genetic rodent model of midfacial hypoplasia in which airway obstruction has been assessed. It thus presents as an important model to study the etiology and systemic consequences of midfacial hypoplasia. Findings from this study will serve as a foundation to better understand the development and management of pediatric airway obstruction in humans.

## RESULTS

### Loss of *Bmp7* leads to midfacial hypoplasia and early lethality

**Fig. 1.**
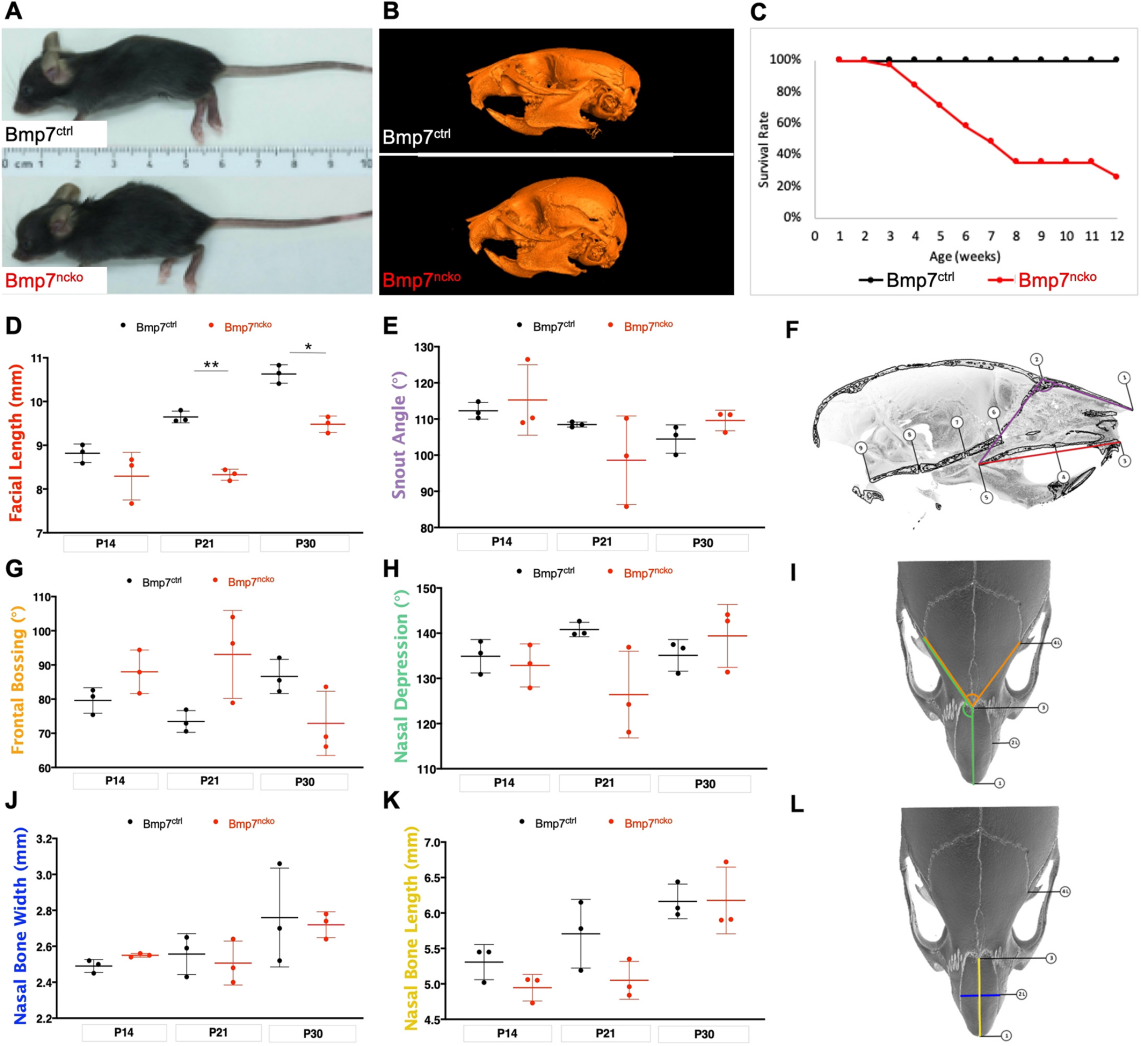
**Bmp7^ncko^ mice present with midfacial hypoplasia at 2 weeks,** **which persists with age.** (A) Overview of Bmp7 control (Bmp7^ctrl^) and Bmp7 mutant (Bmp7^ncko^) mice. (B) Micro-computed tomography (μCT) reconstruction of 1-month-old mouse skulls. Bmp7^ncko^ skull has a distinctive dished midface compared with that of Bmp7^ctrl^. (C) Survival rate plotted against mouse age, indicating marked decrease in the survival of Bmp7^ncko^ mice beginning at 3 weeks, with only 30% of Bmp7^ncko^ mice surviving past 8 weeks (*n*=30). (D) Cross-sectional longitudinal morphometric measurement of facial length of Bmp7^ctrl^ (black) and Bmp7^ncko^ (red) skull dimensions at 2 weeks of age (P14), 3 weeks (P21) and 1 month (P30), indicating a shorter facial length in the mutant mice. Landmarks used for this measurement are depicted in F in red. (E) Snout angle, depicted in F in purple. (F) Midsagittal representation of landmarks used for measurements taken in D and E. (G) Frontal bossing angle, depicted in I in orange. (H) Average of left and right nasal depression angles, depicted unilaterally in I in green. (I) Superior view diagram of measurements taken in G and H. (J) Nasal bone width, depicted in L in blue. (K) Nasal bone length, depicted in L in yellow. (L) Diagram of measurements taken in J and K. Superior view of the skull. For all measurements, *n*=3/genotype/age. *P<0.05, **P<0.01 (two-tailed independent unpaired Student's *t*-test). Anatomical description of the landmarks is shown in Fig. S1.

Bmp7^ncko^ mice appeared inconspicuous during the first 2 weeks of life. From ∼2 weeks of age, a midfacial depression was observed in the mutants to a varying degree (Fig. 1A,B). Starting at ∼3 weeks of age, a failure to thrive with variable onset was observed that ultimately led to death, with only 30% of mutant mice surviving past 8 weeks (Fig. 1C). To determine whether the midfacial depression/hypoplasia is associated with the mortality, a morphometric assessment of the craniofacial complex was conducted on 2-week-old [postnatal day (P)14], 3-week-old (P21) and 4-week-old (P30) mice using a landmark-based approach (Wei et al., 2017). A detailed description of the landmarks used can be found in Fig. S1. The difference in facial length (Fig. 1F) between Bmp7^ncko^ and control mice only became significant at P21 (Fig. 1D) (*P*=2.40×10^–04^) as a consequence of reduced facial growth between the 2- and 3-week time points. Although craniofacial growth catches up subsequently, the difference still persisted at P30 (*P*=2.10×10^–03^). Other craniofacial characteristics such as snout angle (Fig. 1E,F) frontal bossing (Fig. 1G,I), nasal depression (Fig. 1H,I), nasal width (Fig. 1J,L) and length (Fig. 1K,L) showed no significant differences in this limited cohort of mice. The *P*-values for these measurements are provided in Table S1. This morphometric analysis establishes that deletion of *Bmp7* from neural crest cells leads to midfacial hypoplasia that becomes prominent around P21.

### Bmp7^ncko^ mice develop craniofacial abnormalities commonly observed in children with midfacial hypoplasia

**Fig. 2.**
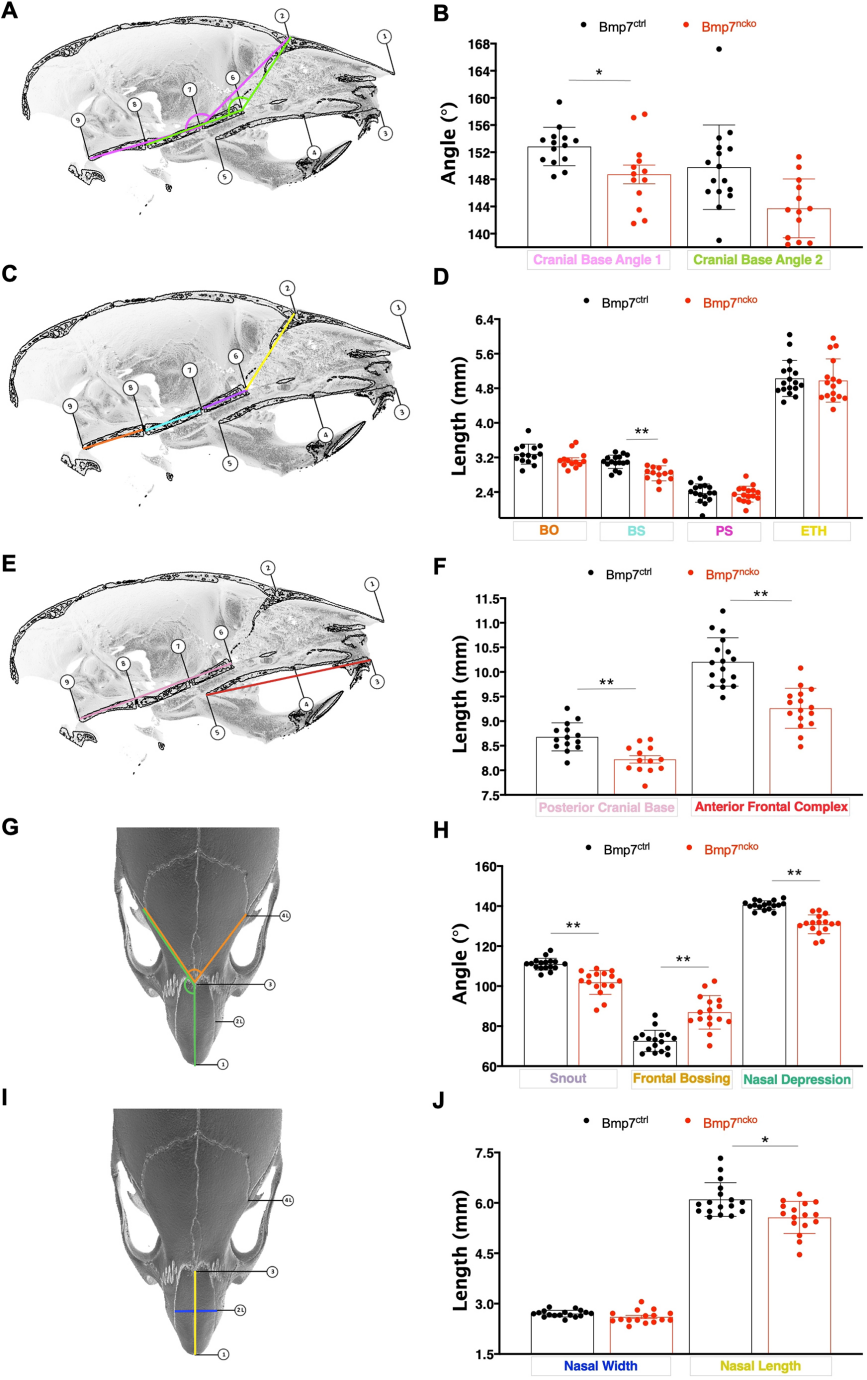
**Craniofacial growth defects contributing to midfacial hypoplasia in 1-month Bmp7^ncko^ mice.** The angle (A,B) and lengths (C,D) of the individual components forming the cranial base were measured using landmarks placed on mid-sagittal cross-sections of the mouse skulls. (A,B) Angles of cranial base 1 (pink) (*n*=27) and cranial base 2 (green) (*n*=29) (A) demonstrated an acute-angled cranial base in Bmp7^ncko^ mice with *P*-values of 0.05 and 0.23, respectively (B). (C) Landmarks used to measure lengths of the BO, BS, PS and ETH are depicted in orange, cyan, violet and yellow, respectively. (D) Measurements indicated a reduction in length of the BS in the Bmp7^ncko^ mice (BO, *n*=27, *P*=0.11; BS, *n*=29, *P*<0.001; PS, *n*=32, *P*=0.76; ETH, *n*=33, *P*=0.76). (E) Landmarks of the posterior cranial base and anterior frontal complex, depicted in pink and red, respectively (compare to Fig. 1F). (F) Measurements demonstrate a significant decrease in both posterior cranial base (*n*=27, *P*<0.001) and anterior frontal complex (*n*=33, *P*<0.001). (E,G) Angles of snout (E), frontal bossing (G, compare to Fig. 1I) and nasal depression (G), depicted in purple, orange and green, respectively. (H) Bmp7^ncko^ mice demonstrate more acute snout angle, increased frontal bossing and more acute angle of nasal depression (*n*=33, *P*<0.001). (I) Nasal bone width and length, depicted in blue and yellow, respectively (compare to Fig. 1L). (J) Measurements of nasal width and length suggest that the mutant mice have a decreased nasal bone length (*n*=33, *P*<0.05), with no changes to nasal width (*n*=33, *P*=0.07). BO, basioccipital bone; BS, basisphenoid bone; PS, presphenoid bone; ETH, ethmoid bone. *P<0.05, **P<0.001 (two-tailed independent unpaired Student's *t*-test). Anatomical description of the landmarks is shown in Fig. S1.

We next performed a detailed morphometric analysis on a larger cohort of 4-week-old control (Bmp7^ctrl^) and Bmp7^ncko^ mice to test for additional craniofacial alterations commonly observed in children with midfacial hypoplasia and upper-airway obstruction (Cielo et al., 2016). Landmark-based morphometrics (for details, see Fig. S1) identified a more acutely angled cranial base (*P*=1.7×10^–02^; Fig. 2A,B) with a significantly shorter basisphenoid (*P*=3.58×10^–06^; Fig. 2C,D). No significant changes were observed in the length of the basioccipital, presphenoid and ethmoid bones (Table S2). Assessment at earlier time points (P14, P21) showed no significant changes (Table S3). Significant length reductions were found both for the posterior (*P*=2.44×10^–05^) and anterior (*P*=1.18×10^–06^) frontal complex (Fig. 2E,F). Changes to snout angle (Fig. 2G,H), frontal bossing angle (Fig. 2G,H) and nasal depression angle (Fig. 2G,H), along with nasal width (Fig. 2I,J) and length (Fig. 2I,J), are a reflection of the significantly more acute-angled snout (*P*=1.43×10^–05^), depressed nasal bones (*P*=3.21×10^–07^) and increased frontal bossing (*P*=4.84×10^–06^) in 1-month-old Bmp7^ncko^ mice. Additionally, the length (*P*=3.80×10^–03^), but not the width, of the nasal bones was significantly reduced. Overall, these findings are consistent with features of midfacial hypoplasia and reduced upper-airway space.

**Fig. 3.**
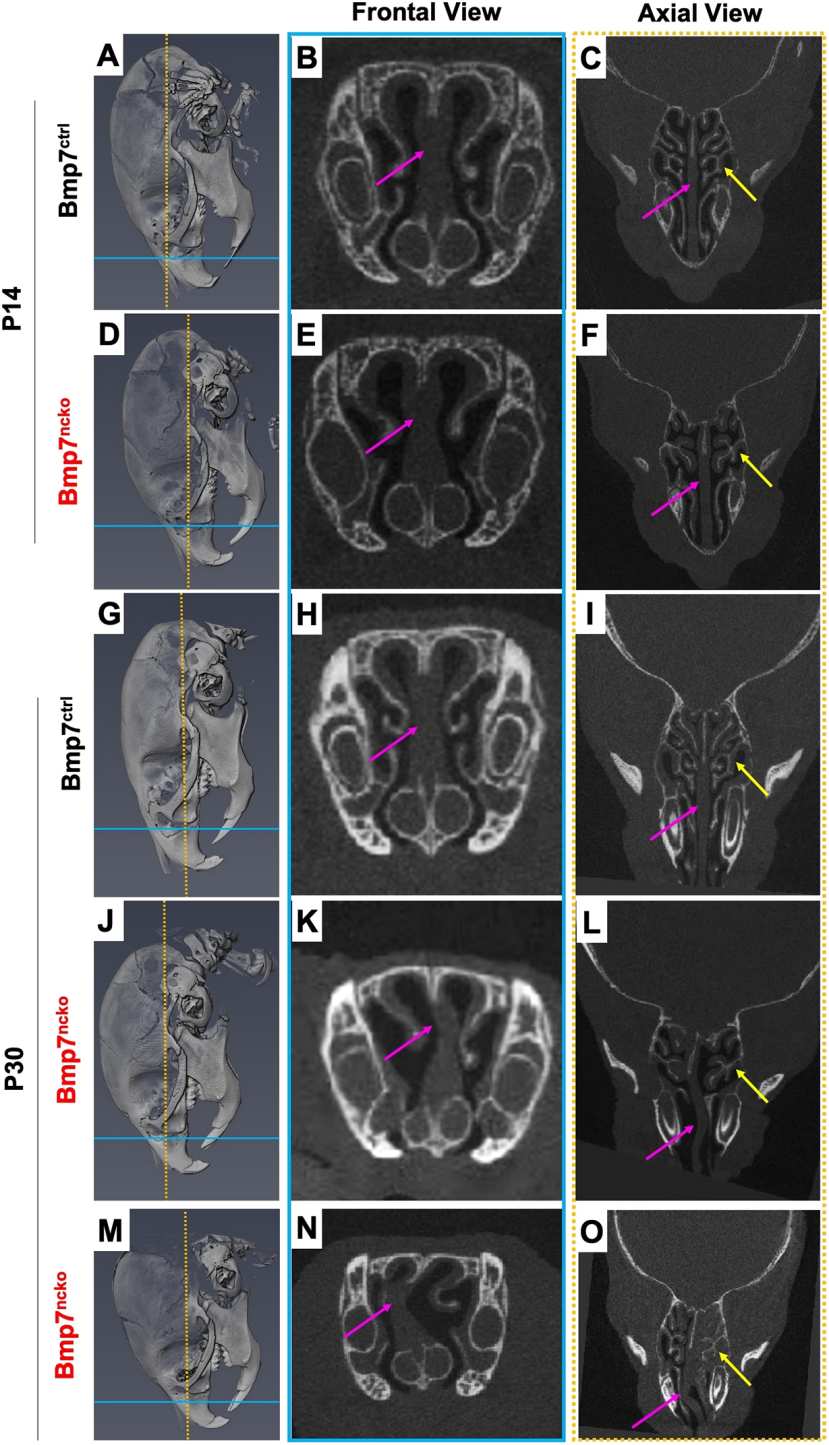
**Bmp7^ncko^ mice present with nasal septum deviation and turbinate dysfunction.** (A-O) μCT representation of frontal (blue line) and axial (orange dotted line) planes used to characterize nasal septum and turbinate abnormality in 2-week-old (P14) (A,D) and 1-month-old mice (P30) (G,J,M). At P14, mice present no structural differences in the nasal septum (pink arrows) between control (B,C) and mutant (E,F) mice under both views. However, abnormal turbinate branching and swelling of mucosa surrounding the turbinates (yellow arrows) was already observed in the mutant mice at 2 weeks (F). At P30, all mutant mice present with nasal septum deviation in both frontal (K,L) and axial (N,O) views in comparison to the control mice (H,I). However, the degree of nasal septum deviation varies between each mutant (K versus N). Similarly, turbinate branching dysfunction and swelling of the mucosa persists in the mutant mice (L-O) at P30 as well. Frontal views of the nasal cavity are outlined in blue lines and axial views in orange dotted lines. *n*=3/age/genotype.

In addition to changes to cranial base length and angle, two other craniofacial differences are commonly observed in children with midfacial hypoplasia and upper-airway obstruction: nasal septum deviation and turbinate hypertrophy (Milanesi et al., 2019). To characterize potential changes to nasal septum and turbinates, frontal (blue lines, Fig. 3) and axial (orange dotted lines, Fig. 3) planes were identified from µCT scans of P14 and P30 mice skulls. The nasal septum (pink arrows, Fig. 3) in P14 Bmp7^ctrl^ (Fig. 3A-C) and Bmp7^ncko^ mice (Fig. 3D-F) showed no obvious differences on both frontal and axial planes. However, Bmp7^ncko^ mice showed reduced turbinate branching (yellow arrows) and their turbinates appeared larger (Fig. 3F). At P30, all Bmp7^ncko^ mice had developed nasal septum deviation (pink arrows) (Fig. 3J-O) compared to controls (Fig. 3G-I). The extent, shape, direction and location, as well as the degree of deviation was variable (Fig. 3K,N). Turbinate development appeared disturbed. Reduced branching and ossification as well as slight swelling of turbinate soft tissue were apparent bilaterally at P14. This swelling and reduced turbinate branching was even more noticeable at P30 (Fig. 3I,L,O). Both nasal septum deviation and turbinate swelling can impede airflow in the nasal cavity, thus potentially exacerbating any other physical airway obstruction.

### Evidence of spontaneous apneas and post-sigh apneas in Bmp7^ncko^ mice

**Fig. 4.**
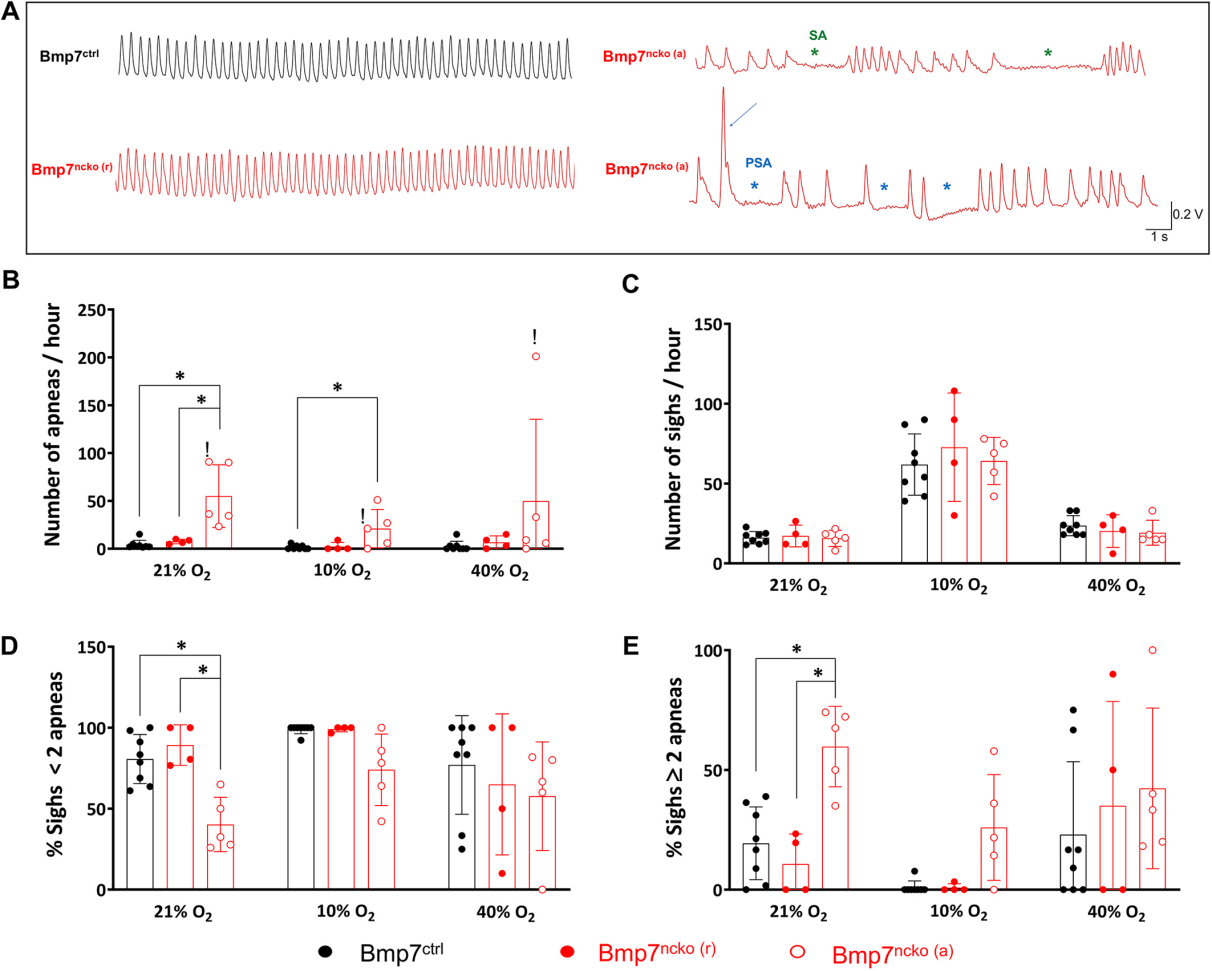
**Spontaneous apnea, sigh and post-sigh apnea during normoxia, hypoxia and hyperoxia in Bmp7^ctrl^, Bmp7^ncko (r)^ and Bmp7^ncko (a)^ mice.** (A) Representative traces showing normal breathing pattern in Bmp7^ctrl^ (black trace) and Bmp7^ncko (r)^ mice (bottom-left red trace); breathing pattern with spontaneous apneas (SA) (green asterisks) in Bmp7^ncko (a)^ mice, and sigh (blue arrow) followed by three apneas (PSA; blue asterisks) in Bmp7^ncko (a)^ mice. (B,C) Number of apneas/h (B) and number of sighs/h (C) in the three experimental groups during normoxia (21% O_2_), hypoxia (10% O_2_) and hyperoxia (40% O_2_). (D,E) Percentage (%) of sighs with <2 apneas (D) and % of sighs with ≥2 apneas (E) in the three experimental groups during normoxia (21% O_2_), hypoxia (10% O_2_) and hyperoxia (40% O_2_). *P<0.05 (two-way repeated measures ANOVA and Holm-Sidak post test). The exclamation marks indicate outliers. Bmp7^ncko (r)^, mutant mice with no changes to regular breathing; Bmp7^ncko (a)^, Bmp7^ncko^ mice with SA.

Whole-body plethysmography was used to assess breathing patterns in Bmp7^ncko^ mice. Eight control and ten Bmp7^ncko^ mice were measured at a time when the mutant mice had developed nasal septum deviation (4-6 weeks of age). Of the ten Bmp7^ncko^ mice analyzed in this study, five elicited a greater number of spontaneous apneas (SAs) in normoxia compared to Bmp7^ctrl^ mice (*P*=4.00×10^–03^) and the remaining Bmp7^ncko^ mice (*P*=2.00×10^–02^; Fig. 4A,B). Consequently, we divided the mutant mice cohort into two groups for all respiratory analysis: Bmp7^ncko (r)^ (regular breathing) and Bmp7^ncko (a)^ (high-frequency SA).

Movie 1 shows recordings of plethysmography traces associated with the behavior of a Bmp7^ctrl^ mouse during eupneic breathing, and a Bmp7^ncko (a)^ mouse experiencing SA. The presence of greater number of SAs in the Bmp7^ncko (a)^ mice in comparison to Bmp7^ctrl^ mice persisted during hypoxia (*P*=0.01) as well as during the first minute of recovery to normoxia (*P*=5.00×10^–03^; Bmp7^ctrl^, 0.3±0.5; Bmp7^ncko (r)^, 3.0±4.7; Bmp7^ncko (a)^, 8.2±5.5). The presence of a greater number of SAs in the Bmp7^ncko (a)^ group in comparison to the Bmp7^ctrl^ and Bmp7^ncko (r)^ groups disappeared during hyperoxia (40% O_2_) (*P*=0.21 and *P*=0.54, respectively; Fig. 4B). However, there was one animal that appeared to be an outlier, so it was excluded from the statistical analysis for all the gas exposures. These results indicate that, during normoxic and hypoxic conditions, apneas are more frequent in a subpopulation of Bmp7^ncko^ mice and this difference was eliminated in hyperoxia.

Sigh frequency was comparable between all experimental groups. As expected (Li et al., 2016), hypoxia exposure induced an increased number of sighs in all groups (*P*<0.001) compared to normoxia (Fig. 4C). Under physiological conditions, sighs may be followed by brief apneas [post-sigh apneas (PSAs)] or respiratory irregularities (Bastianini et al., 2019). Short respiratory disruptions following a sigh (≤1 apnea) was reduced in the Bmp7^ncko (a)^ group compared to the Bmp7^ctrl^ and Bmp7^ncko (r)^ groups (*P*<0.01; Fig. 4D), whereas prolonged PSA events (≥2 apneas following a sigh, as shown in Fig. 4A) increased (*P*<0.001; Fig. 4E).

### Bmp7^ncko^ mice with apneas show altered baseline respiratory, metabolic and thermoregulatory patterns

**Fig. 5.**
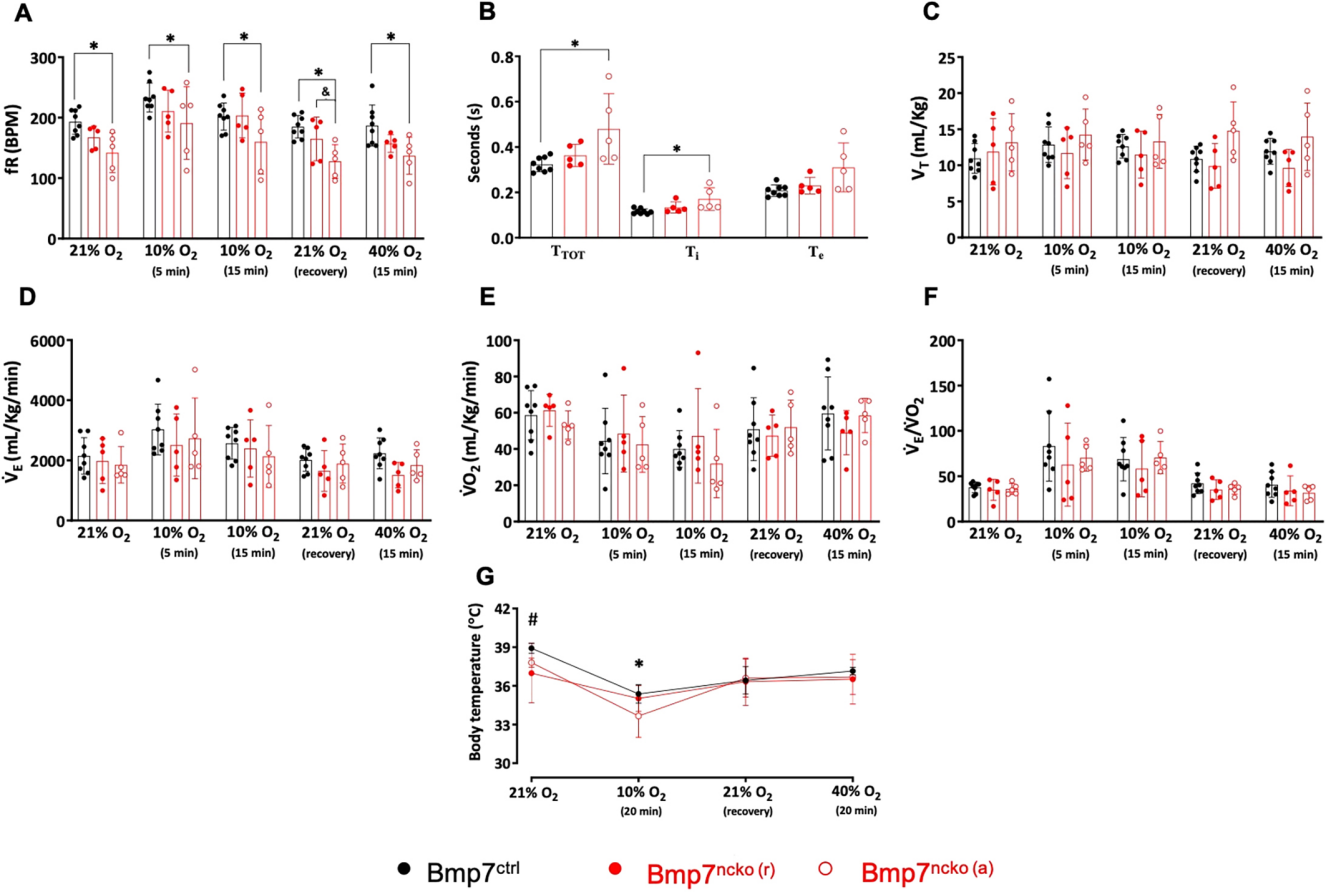
**Respiratory and metabolic patterns during normoxia, hypoxia and hyperoxia in Bmp7^ctrl^, Bmp7^ncko (r)^ and Bmp7^ncko (a)^ mice.** (A) Respiratory frequency (fR) in the three experimental groups during normoxia (21% O_2_), 5 min and 15 min of hypoxia (10% O_2_), recovery to baseline conditions (21% O_2_) and 15 min of hyperoxia (40% O_2_). (B) Breath cycle duration (T_TOT_), inspiratory time (T_i_) and expiratory time (T_e_) during normoxia in the three experimental groups. (C-F) Tidal volume (V_T_) (C), minute ventilation (*V̇*_E_) (D), O_2_ consumption (*V̇*_O_2__) (E) and air convection requirements (*V̇*_E_/*V̇*_O_2__) (F) in the three experimental groups during normoxia (21% O_2_), 5 min and 15 min of hypoxia (10% O_2_), recovery to baseline conditions (21% O_2_) and 15 min of hyperoxia (40% O_2_). (G) Body temperature (Tb) in the three experimental groups during normoxia (21% O_2_), 20 min of hypoxia (10% O_2_), recovery to baseline conditions (21% O_2_) and 20 min of hyperoxia (40% O_2_). *P<0.05 for Bmp7^ctrl^ versus Bmp7^ncko (a)^ mice; ^#^*P<*0.05 for Bmp7^ctrl^ versus Bmp7^ncko (r)^ mice; ^&^*P*<0.05 for Bmp7^ncko^ (r) versus Bmp7^ncko (a)^ (two-way repeated measures ANOVA and Holm-Sidak post test, except one-way ANOVA for B). Bmp7^ncko (r)^, mutant mice with no changes to regular breathing; Bmp7^ncko (a)^, Bmp7^ncko^ mice with apneas. *n*=8 control mice, *n*=10 Bmp7^ncko^ mice.

Along with a greater number of SAs and longer PSA events, Bmp7^ncko (a)^ mice had a lower baseline respiratory frequency (fR) (*P*=1.60×10^–02^; Fig. 5A) and an increase in cycle duration of each respiratory event (T_TOT_) (*P*=4.10×10^–02^) owing to an increase in the inspiratory time (T_i_) (*P*=1.90×10^–02^) with a trend to increase in the expiratory time (T_e_) (*P*=7.90×10^–02^; Fig. 5B). Noteworthy, the two Bmp7^ncko (a)^ mice with greater SA number showed greater T_TOT_, T_i_ and T_e_ compared to the other mice from the same group. Bmp7^ncko (r)^ mice showed a trend towards reduced values for fR compared to control mice; however, the differences were not significant. Despite fR changes, baseline tidal volume (V_T_) and minute ventilation (*V̇*_E_), a measure of the total volume of gas inhaled per minute, did not differ between the groups (Fig. 5C,D, respectively). Despite the greater number of SAs in the Bmp7^ncko (a)^ mice, we did not find changes in variability of respiratory parameters (Table S5), or normoxic O_2_ consumption (*V̇*_O_2__) and air convection requirements (*V̇*_E_/*V̇*_O_2__) (Fig. 5E,F), among the groups. Overall baseline body temperature (Tb) was lower in the Bmp7^ncko (r)^ mice compared to that in the control mice (*P*=1.20×10^–02^; Fig. 5G), mainly due to greater Tb individual variability within the Bmp7^ncko (r)^ mice.

To establish the timeline of when Bmp7^ncko^ mice develop respiratory disturbances, a separate plethysmography assessment was conducted at 2 weeks of age, prior to the development of nasal abnormalities. At this time, the numbers of SAs and PSAs were comparable between the Bmp7^ncko^ and Bmp7^ctrl^ mice (Fig. S2). However, the Bmp7^ncko^ mice demonstrated a trend towards lower fR and an increase in T_TOT_ as a result of an increase in T_i_ and T_e_. These results suggest that the majority of respiratory disturbances develop following the development of craniofacial abnormalities.

### Ventilatory and metabolic responses to hypoxia and hyperoxia

The same mice used for breathing analysis in normoxia were exposed to acute hypoxia (10% O_2_) and hyperoxia (40% O_2_) to evaluate their respiratory responses to different levels of oxygen. In the control group, we observed the biphasic ventilatory response, in which hypoxia induced hyperventilation as shown by an increase in *V̇*_E_ and *V̇*_E_/*V̇*_O_2__ in the first 5 min of exposure (*P*<0.001; Fig. 5D,F, respectively), followed by an attenuation at 15 min, with no significant difference from baseline values. Significant increase in fR was observed both at 5 min (*P*<0.001) and 15 min (*P*<0.001; Fig. 5A; Fig. S2). Hypoxia also caused a decrease in *V̇*_O_2__ (*P*<0.05; Fig. 5E) and body temperature (*P*<0.001; Fig. 5G). A comparable physiological response to hypoxia was observed in both the Bmp7^ncko (a)^ and Bmp7^ncko (r)^ groups (*P*<0.05). Even though Bmp7^ncko (a)^ had a lower breathing frequency in normoxic conditions, the hypoxic frequency response shown as a percentage of baseline was similar among the three groups (Fig. S3). However, mice from the Bmp7^ncko (r)^ group showed a variable hypoxic ventilatory response. Two mice showed hyperventilation, as seen by larger values in the *V̇*_E_/*V̇*_O_2__ data (Fig. 5F), whereas the other three mice showed no change in *V̇*_E_/*V̇*_O_2__ compared to the values in normoxia. It is noteworthy that the one Bmp7^ncko^ mouse in which *V̇*_O_2__ did not drop during hypoxia showed a baseline body temperature of 33°C, which is below the normal range, and no further decrease during hypoxia was observed. Therefore, it is legitimate to deduct that thermoregulation in this specific mouse seemed compromised, potentially further affecting ventilation and O_2_ consumption modulation. Interestingly, all Bmp7^ncko (r)^ mice showed an increased fR induced by hypoxia at 5 min that persisted throughout the hypoxic exposure (*P*<0.01) (Fig. 5A), although no changes were observed for the other physiological variables (*V̇*_E_, V_T_, *V̇*_O_2__ and *V̇*_E_/*V̇*_O_2__).

Hyperoxia did not evoke any changes in the respiratory, metabolic or thermoregulatory variables within and between experimental groups, with the exception of a lower fR in Bmp7^ncko (a)^ mice during 40% O_2_ (*P*=5.00×10^–03^), owing to the lower baseline fR.

### Reduced delta oxygen and upregulation of hydroxyproline observed in Bmp7^ncko^ mice

**Fig. 6.**
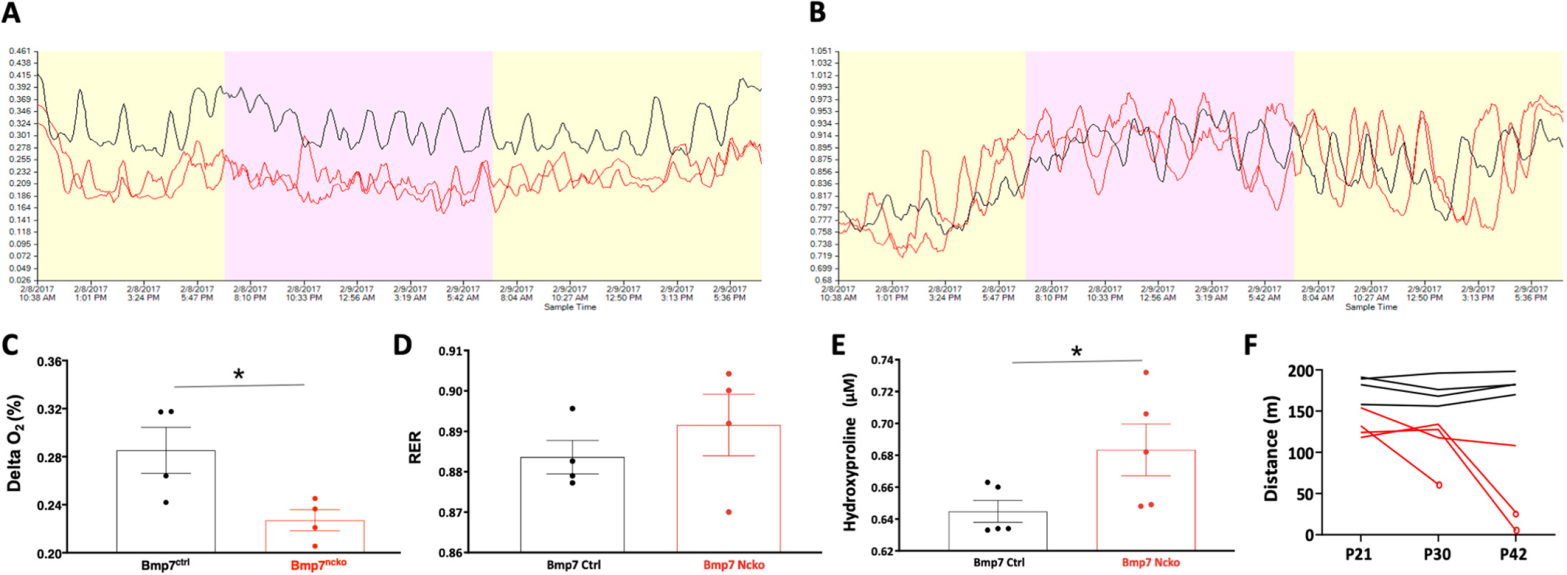
**Oxygen consumption is reduced in the Bmp7^ncko^ mice.** One-month old Bmp7^ctrl^ and Bmp7^ncko^ mice were measured in the Comprehensive Lab Monitoring System (CLAMS) chamber for oxygen consumption and respiratory exchange. (A,C) Representative trace of oxygen consumption (delta oxygen) plotted against time for one control (black) and two Bmp7^ncko^ mice (red) (A) demonstrated a reduction in oxygen consumption (C) (*n*=4, *P*<0.05). (B,D) Representative trace of respiratory exchange ratio (RER) plotted against time for one control (black) and two Bmp7^ncko^ mice (red) (B) demonstrated no significant changes (D) (*n*=4, *P*=0.39). (E) Metabolite analysis of blood serum collected from Bmp7^ctrl^ and Bmp7^ncko^ mice demonstrated a significant upregulation in hydroxyproline concentration in the mutant mice (*n*=5, *P*<0.05). (F) Exhaustion treadmill test on Bmp7^ctrl^ and Bmp7^ncko^ mice revealed a reduction in physical activity in the mutant mice (red) as mice age. Circles denote mutant mice that became severely fatigued and had to be euthanized. *P<0.05 (two-tailed independent unpaired Student's *t*-test).

Four-week-old Bmp7^ctrl^ and Bmp7^ncko^ (*n*=5/genotype) mice were placed individually in CLAMS chambers for 24 h to measure oxygen consumption and respiratory exchange. Delta oxygen, the difference in oxygen fraction in the inflow and outflow of the chamber, indicating oxygen consumption, was reduced in Bmp7^ncko^ mice, as shown by representative traces of one control and two Bmp7^ncko^ mice (Fig. 6A). The trace of the respiratory exchange ratio (RER), which is the rate of carbon dioxide production to the rate of oxygen consumption, showed no clear differences (Fig. 6B). Quantification of the delta oxygen (Fig. 6C) and RER (Fig. 6D) demonstrated a significant reduction in oxygen consumption (*P*<0.05) and no change in RER in the Bmp7^ncko^ mice. To understand the systemic consequences, blood serum from P30 Bmp7^ctrl^ and Bmp7^ncko^ mice (*n*=5/genotype) was collected and screened for changes to metabolite concentrations using an unbiased metabolomics assay. In each sample, 154 metabolites were detected and quantified. A significant upregulation of hydroxyproline (*P*<0.05), an indicator of collagen synthesis or release, was identified in Bmp7^ncko^ mice (Fig. 6E). This demonstrated that Bmp7^ncko^ mice consume less oxygen over time, and that it is possible to identify metabolite differences between control and mutant mice.

### Bmp7^ncko^ mice show decreased exercise capacity at P30

To test whether Bmp7^ncko^ mice develop reduced endurance for physical activity, mice were assessed on a treadmill longitudinally starting at the age of 3 weeks. Physical activity in the mice at 3 weeks was comparable between the groups, with an increasing reduction in activity after every subsequent assessment (Fig. 6F). The difference in physical ability was confirmed in an acute speed test, in which Bmp7 mutant mice tired very quickly and spent more time on the resting plate, indicating that they do not have the same capacity for physical exercise (Movie 2). Thus, craniofacial defects and associated breathing disturbances might lead to a reduced ability for physical activity, possibly due to airway obstruction, in this rodent model.

## DISCUSSION

In this study, Bmp7^ncko^ mice demonstrated structural and growth abnormalities in the craniofacial complex, alteration in breathing pattern, i.e. apnea events and lower breathing frequency, and reduction in the capacity for physical exercise. Thus, the Bmp7^ncko^ mouse model emulates several features often linked to the onset of airway obstruction in pediatric OSA (Cielo et al., 2016), including midfacial hypoplasia, shorter cranial base and nasal septum deviation. By delineating the sequence of craniofacial changes, we show that they tend to precede the onset of respiratory disturbances. We also show how this model might be used to identify systemic manifestations caused by upper-airway obstruction.

Despite the abundance of genetic rodent models of midfacial abnormalities, to our knowledge, no rodent model of nasal airway obstruction, SRBD and midfacial deficiency has been described. Such a model could greatly benefit the field of pediatric sleep medicine. Most rodent models of SRBD and/or airway obstruction address only isolated facets of this complex disorder (Table 1). Animals exhibiting IH have most commonly been used to study the effects of obstruction (Fletcher et al., 1992; Gozal et al., 2001). However, there is controversy in whether the settings used to mimic IH in rodent experiments are clinically translatable to IH experienced in humans (Farré et al., 2018). Mechanical obstruction of the airways or oxygen deprivation have also been used (Almendros et al., 2011; Tarasiuk and Segev, 2018). The SRBD/OSA rodent models with ‘naturally occurring’ airway obstruction, such as the Zucker hooded rat (Iida et al., 1996), exhibit loss of airway patency owing to excessive soft tissue or obesity. Understanding how abnormalities of the craniofacial complex lead to airway obstruction and SRBD has been hampered by the fact that models with severe congenital craniofacial anomalies, which often involve a cleft palate, die at birth, preventing postnatal analysis (Hong and Krauss, 2012; Perlyn et al., 2006). On the other hand, models that show snout asymmetries, as for instance often observed in *Bmp7* haploinsufficient mice, have not been assessed for breathing abnormalities or show early lethality as a consequence, thus making the Bmp7^ncko^ model unique. It is the first model to describe how craniofacial abnormalities such as midfacial hypoplasia, short and acute-angled cranial base and nasal septum deviation predispose mice to nasal airway obstruction postnatally. A shorter cranial base is often associated with premature fusion of cranial base synchondrosis (Rosenberg et al., 1997). Careful examination revealed that this was not the case in Bmp7^ncko^ mice. It is therefore possible that the shorter cranial base is a consequence of reduced growth itself. Bmp7^ncko^ mice develop midfacial hypoplasia during the time of rapid midfacial growth (2-3 weeks of age). Midfacial hypoplasia on its own appears to be insufficient to induce upper-airway obstruction. Hypoplasia and associated shorter and more acute-angled cranial base and reduced nasal bone length (Figs 1 and 2) are all established prior to nasal airway obstruction. Only once nasal septum deviation and turbinate swelling are observed around 4 weeks do the animals start developing respiratory disturbances. The hypopneas (lower fR), an increase in T_TOT_ and T_i_, and increased number of SA and PSA irregularities in a subpopulation of the Bmp7^ncko^ mice are features likely to be associated with nasal airway obstruction owing to the combination of craniofacial abnormalities. Little was known about the sequence, etiology and consequences of these different craniofacial anomalies. Craniofacial characteristics have been described in detail for several mouse strains with midfacial hypoplasia (Eimar et al., 2016; Marulanda et al., 2017; Suzuki et al., 2016; see also Table 1). None of these models show nasal septum deviation and/or nasal airway obstruction, supporting the notion that midfacial hypoplasia on its own does not necessarily lead to nasal airway obstruction (Finkelstein et al., 2000; Tan et al., 2016). The survival plot (Fig. 1C) indicates that the majority of Bmp7^ncko^ mice die by the age of 8-12 weeks, which coincides with the plateauing of craniofacial growth (Vora et al., 2015). Although challenging to assess in detail, we believe that cumulative craniofacial changes either fatally affect mice by that age, or will never be sufficiently severe, allowing mice to survive long term.

**Table 1.**
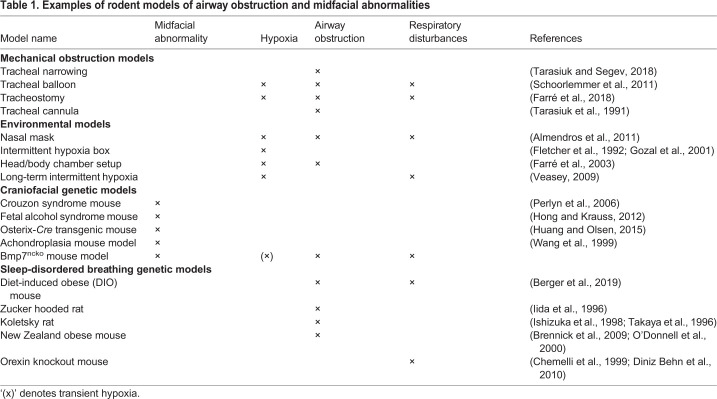
**Examples of rodent models of airway obstruction and midfacial abnormalities**

Similarly, when comparing pediatric OSA cohorts of different ethnicities, differences in the prevalence of craniofacial abnormalities were noted (Lee et al., 2010; Tan et al., 2016). Those ethnicities have distinct craniofacial features with different degrees of sagittal, vertical and transversal maxilla-mandibular interrelations. It was also observed that nasal septum deviation, turbinate hypertrophy and enlarged adenoids greatly increase the prevalence of upper-airway obstruction in children with midfacial hypoplasia (Clark et al., 2018; Pacheco et al., 2015; Tan et al., 2016). Turbinate hypertrophy and deviated nasal septum frequently occur together (Baddam et al., 2020; Hsu and Suh, 2018), but again their presence on their own is insufficient to predict upper-airway obstruction (Baddam et al., 2020). In Bmp7^ncko^ mice, turbinate swelling precedes nasal septum deviation. Currently, the cause of turbinate swelling is unclear. Although abnormal turbinate development can be observed, the swelling could also be a consequence of abnormal mucus drainage, cellular changes to turbinate mucosa and/or altered airflow.

Although all Bmp7^ncko^ develop nasal septum deviation at 4 weeks, 50% of these mice developed SA between 4 and 6 weeks. This is suggestive that Bmp7^ncko^ mice experience respiratory distress due to airway obstruction to varying degrees. Here, we showed evidence that morphological changes in the upper airways disrupt regular breathing pattern, possibly due to airway obstruction. Increased inspiratory airway resistance may lead to two mechanisms to maintain ventilation: (1) an increase in inspiratory muscle drive; and (2) an increase in the T_i_, to achieve a comparable V_T_ over an expanded T_i_ (Hernandez et al., 2012; Mann et al., 2017). In the present study, Bmp7^ncko (a)^ mice showed an increased T_TOT_, mainly due to an increase in the T_i_ and a tendency for a prolonged T_e_ compared to control mice, resulting in no changes in V_T_ and *V̇*_E_. In patients with OSA, when these adjustments in breath timing do not compensate ventilation, apneas or hypopneas may occur (Gleadhill et al., 1991; Sanders and Moore, 1983; Yamauchi et al., 2011), as we have observed in a subpopulation of Bmp7^ncko^ mice. Indeed, adaptation to mouth breathing has been observed in children with upper-airway obstruction (Trosman and Trosman, 2017). Although mice are described as obligate nose breathers (Harkema and Morgan, 1996), adaptation to mouth breathing (Agrawal et al., 2008; Niaki et al., 2008) or gasping (Khurana and Thach, 1996) has been observed in the event of nasal obstruction. Assuming that adaptation occurs in Bmp7^ncko^ mice, further experiments will be necessary to distinguish adaptation to mechanical and/or central control of breathing.

Along with a greater SA incidence, Bmp7^ncko (a)^ mice also present with a greater frequency of irregular breathing following a sigh. The mechanisms underlying apneas and, particularly, PSAs are not completely understood. They may involve inhibition of inspiration through activation of pulmonary stretch receptors, the Breuer–Hering reflex, or decreased drive to breathe due to lower CO_2_ levels induced by the sigh (Bastianini et al., 2019; Davis and O'Donnell, 2013; Saito et al., 2002). Bmp7^ncko (a)^ mice may have alteration in the vagal reflex, or compromised central responses to the reflex, leading to more apneic events, which could also explain the lower breathing frequency. Derivatives of neural crest contribute to the carotid body and sensory neurons (Annese et al., 2017; Pavan and Raible, 2012), both of which provide important excitatory signals for central breathing control, especially in hypoxic conditions. Bmp7 could affect the development of either structure. Although a role for Bmp7 in neurogenesis is well established (Choe et al., 2013; Segklia et al., 2012), nothing is known about neural crest-derived Bmp7 contribution to carotid body development and function. It should be noted that the Wnt1-Cre driver used in this study has been shown to affect brain development (Lewis et al., 2013). For this reason, *Bmp7^fx/+^*;Wnt1-Cre mice were additionally assessed for craniofacial changes. There was no evident septum deviation or early lethality, as seen in the Bmp7^ncko^ mice. Therefore, these mice were not subjected to functional plethysmography.

In the present study, both Bmp7^ctrl^ and Bmp7^ncko^ mice showed a biphasic ventilatory response to hypoxia (Moss, 2000; Teppema and Dahan, 2010), with a decrease in *V̇*_O_2__ and body temperature (Peña and Ramirez, 2005), indicating that peripheral chemosensitivity and centrally regulated breathing control are most likely intact. However, the drop in Tb was greater in the Bmp7^ncko (a)^ mice compared to controls, suggesting that the thermoregulatory control to hypoxia may be affected by deletion of *Bmp7* in the neural crest.

Supplemental oxygen therapy has been examined to have effects on the pathophysiology of OSA, in humans, although its benefits are controversial (Mehta et al., 2013; Wellman et al., 2008; Xie et al., 2013). We wanted to test whether, after reducing the ventilatory drive with hyperoxia exposure, Bmp7^ncko (a)^ mice would show changes in breathing pattern or irregularities. Hyperoxia did not affect the number of PSAs or cause any dysfunction in ventilation. However, it reduced the number of SAs in the Bmp7^ncko (a)^, except for one mouse in which hyperoxia potentiated the number of SAs. These results suggest that, at least in our mouse model, hyperoxia may help to reduce the occurrence of SAs in Bmp7^ncko (a)^ mice. However, the fact that one outlier Bmp7^ncko (a)^ mouse showed the opposite effect should not be ignored. The respiratory variability might be a true reflection of the unpredictable response observed in some children using continuous positive airway pressure (Hoffstein et al., 1992).

The trend for lower fR and increased T_i_ and T_e_ in 2-week-old Bmp7^ncko^ mice (Fig. S3), a time prior to structural nasal abnormalities being established, is suggestive that the lack of Bmp7 in the neural crest during embryonic development may affect developmental processes that only later manifest as phenotypic changes. Hence, the structural abnormalities could be a consequence of the inability to repair and recover and grow during the growth spurt (after 3 weeks in mice). Additionally, the understanding that altered cellular and molecular processes in the human body precede structural abnormalities later in life (Gomes et al., 2012) reaffirms the plasticity of the human body, as well as the notion that nasal septum deviation and its consequences establish gradually and not overnight. Thus, better understanding of the underlying genetic alterations associated with nasal airway obstruction will assist in effective management and treatment of the obstruction along with its associated co-morbidities.

The Bmp7^ncko^ model could be used to further investigate the origin of mixed apneas in individuals with craniofacial or neural crest abnormalities. The inability to use craniofacial abnormalities and degree of nasal septum deviation as predictors for the severity of upper-airway obstruction and relative contribution of the different apneas is an accurate reflection of the challenges clinicians face in determining/diagnosing using CT scans whether children with nasal obstruction will develop SRBD and require further intervention (Baddam et al., 2020).

Several biomarkers – such as kallikrein-1, uromodulin, urocortin-3 and orosomucoid-1 – have been proposed as diagnostic markers for pediatric OSA in children, although their sensitivity and specificity have yet to be fully understood (De Luca Canto et al., 2015). Using a limited quantitative metabolomics approach, we tested whether this model can be used to identify potential biomarkers for airway obstruction. From the screening of 154 metabolites, we observed a significant increase in hydroxyproline. This metabolite has been used as an index of total collagen degradation (Krane et al., 1977) but more recently has been directly associated with hypoxia in hepatocellular carcinoma (Tang et al., 2018). In Tang et al. (2018), hydroxyproline was part of the metabolic axis promoting cell survival through modulating hypoxia-inducing factor 1 alpha (HIF1α). Although arterial partial pressure of O_2_ (paO_2_) was not directly measured in our study, the increase in hydroxyproline may suggest, in addition to reduced physical activity, that the Bmp7^ncko^ mice experience episodes of hypoxia. Bmp7^ncko^ mice showed a decrease in the ability to sustain a physical exercise, such as running on a treadmill, with age. This may be an indication of their inability to ventilate efficiently. Similarly, a reduced exercise capacity was also observed in children with OSA (Evans et al., 2014). In Bmp7^ncko^ mice, the nasal airway obstruction is likely to be present both during active and rest phases. OSA has been shown to have wider systemic consequences including bone remodeling (Eimar et al., 2016). A reduction in bone mineral density indicative of changes to bone remodeling has previously been identified in children with OSA (Eimar et al., 2019), but it was not clear whether hypoxia affected bone remodeling or vice versa. Based on our findings, we propose that altered bone metabolism is part of the congenital abnormalities and that hypoxia may be a consequence of altered craniofacial growth.

Our observations on Bmp7^ncko^ mice shed new light on the etiology of nasal airway obstruction as a consequence of insufficient midfacial growth. Midfacial hypoplasia on its own cannot predict or determine the severity of SRBD, highlighting the need to carefully investigate craniofacial abnormalities in children. We find that various anomalies in craniofacial structures synergistically predispose to upper-airway obstruction. The primary reason for these malformations appears to be altered cartilage and bone development. Although the nasal septum, cranial base and nasal bones grow as a developmental unit, the relative contribution of individual components varies even in this genetically defined model. Environmental factors do play a role, although this has not been well studied yet. For this reason, the onset and severity of upper-airway obstruction remain largely unpredictable. Although hypoxic ventilatory response appears largely intact, an increased number of PSA events and alterations in body temperature during normoxia and hypoxia suggest the involvement of the central network for respiratory and thermoregulatory control in the features of the Bmp7^ncko^ mice model. At present, it is not possible to distinguish whether this is a systemic consequence of upper-airway obstruction or due to neural crest-specific roles of Bmp7 outside bone/cartilage. The multifactorial etiology and presence of mixed apneas reflects the challenges clinicians face in treating complex cases of pediatric OSA, warranting an appropriate genetic model for further study. We have shown that this model can be used for the identification of potential predictive biomarkers to help identify which children with craniofacial malformations are likely to develop SRBD.

In summary, this study demonstrates that the Bmp7^ncko^ mouse can be used to identify molecular mechanisms underlying craniofacial abnormalities resulting in nasal airway obstruction to better understand the genetic component contributing to obstruction in children with SRBD.

## MATERIALS AND METHODS

### Animals

All animal experiments were approved by the Research Ethics Office of the University of Alberta (Animal Use and Care Committee; protocol AUP1149), in compliance with guidelines by the Canadian Council of Animal Care. Mouse lines were kept on a C57Bl/6J background at the animal facility of the University of Alberta. *Bmp7^fl/fl^* (Bmp7^ctrl^) mice (Zouvelou et al., 2009) were crossed to Wnt1-Cre mice, Tg(Wnt1-Cre)11Rth, for neural crest-specific deletion of *Bmp7* (*Bmp7^fl/fl^*:Wnt1-Cre) (Bmp7^ncko^). Mice were genotyped using polymerase chain reaction with DNA obtained from tissue biopsies (Zouvelou et al., 2009).

### µCT

For µCT, Bmp7^ctrl^ and Bmp7^ncko^ mice were scanned using MILabs μCT at the School of Dentistry, University of Alberta. The mice were perfused with 4% paraformaldehyde (PFA) and mouse heads were subsequently fixed in 4% PFA for another 24 h prior to scanning. The following parameters were applied for scanning: voltage, 50 kV; current, 0.24 mA; exposure time, 75 ms. Scans were reconstructed at a voxel size of 25 μm. The µCT-based skull reconstructions were used for anatomical comparison of the heads. Landmarks were placed on the skulls and defined angles/lengths were collected to quantify craniofacial differences (Fig. S1). Reconstructed scans were analyzed using AMIRA (Life Technologies) independently by one rater in triplicate, each time placing landmarks anew and collecting measurements.

### Whole-body plethysmography experimental design

Animals were placed individually in the whole-body plethysmography chamber that was initially flushed with room air (21% O_2_) for an acclimation period of 30 to 40 min. *V̇*_E_ and *V̇*_O_2__ were then recorded during baseline (normoxic conditions, 21% O_2_ and N_2_ balance) for 150 min to guarantee that mice would go through the sleep-awake cycle, followed by 20 min exposure to hypoxia (10% O_2_ and N_2_ balance), allowed to recover to normoxia values for 60 min and then exposed to hyperoxia (40% O_2_) for 20 min. Gas mixtures were provided by a gas mixing system (GSM-3 Gas Mixer; CWE Inc., Ardmore, PA, USA). Respiratory and metabolic variables reported represent average values measured during 150 min of normoxia, 0-5 min and 10-15 min of hypoxia, the last 10 min of the recovery period and 10-15 min of hyperoxia. Body temperature was measured before placing the mice into the chamber, 1 min after hypoxia exposure, at 45 min of the recovery period in normoxia and at the end of hyperoxia.

### Measurements of ventilation, O_2_ consumption and body temperature

Ventilation was measured using the open-flow whole-body plethysmography method (Seifert et al., 2000). The mice were placed in a 100 ml or 200 ml chamber, depending on the mouse's body mass, through which gas flow was maintained at rates of 150 ml/min and 300 ml/min, respectively (Lighton, 2008). In this system, the heating and humidification of the air that occurs with rhythmic inspiration and expiration of air causes pressure changes in the chamber that were detected with the differential pressure transducer (Validyne Engineering, Northridge, CA, USA). The pressure signal was acquired using PowerLab System (ADInstruments, Sydney, Australia) and sampled at 200 Hz. Data were analyzed using the data analysis software, LabChart (version 8, ADInstruments). The system was calibrated with injections of 0.25 ml into the plethysmography chamber at the same rate as breathing rate. V_T_ was calculated with the appropriate formula (Drorbaugh and Fenn, 1955): V_T_=V_K_×(P_T_/P_K_)×Tb×(P_B_−P_C_)/Tb×(P_B_−P_C_)−T_A_×(P_B_−P_R_), where P_T_ is the pressure deflection associated with each V_T_, P_K_ is the pressure deflection associated with the injection of the calibration volume (V_K_), T_A_ is the air temperature in the animal chamber, P_B_ is the barometric pressure, P_C_ is the water vapor pressure in the animal chamber, Tb is the body temperature (in Kelvin), and P_R_ is the water vapor pressure at Tb. Ventilation (*V̇*_E_) was calculated as the product of fR and V_T_. P_C_ and P_R_ were calculated indirectly using appropriate tables (Dejours, 1981). All variables were normalized to body mass. Body temperature was measured using a rectal probe (Physiosuite, Kent Scientific, Torrington, CT, USA). Metabolic rate [O_2_ consumption (*V̇*_O_2__)] was measured using pull-mode indirect calorimetry (Cummings et al., 2011; Mortola, 1984). An O_2_ analyzer pump (ADInstruments) was connected to the outflow of the chamber, where subsampled air was dried through a small drying column (Drierite, Sigma-Aldrich, St Louis, MO, USA) before passing to the analyzer. The gas was continuously sampled (1000 Hz) by the O_2_ and CO_2_ analyzers, and the fraction of O_2_ and CO_2_ in the inflow and outflow gas was recorded via PowerLab System/LabChart (version 8). *V̇*_O_2__ was calculated based on the following equation (Depocas and Hart, 1957; Lighton, 2008): *V̇*_O_2__=[FR_e_(FiO_2_–FeO_2_)–FiO_2_(FeCO_2_–FiCO_2_)]/1–FiO_2_, where FR_e_ is the excurrent flow rate, FiO_2_ is the inflow O_2_ fraction, FeO_2_ is the outflow O_2_ fraction, FiCO_2_ is the inflow CO_2_ fraction, and FeCO_2_ is the outflow CO_2_ fraction. *V̇*_O_2__ was normalized to body mass (in kg) and the values were reported under standard conditions of temperature, pressure and dry air.

### SA, sigh and PSA analysis

SAs were defined as at least two missing breath cycles, which correspond to pauses of 1 s or longer in the breathing recordings depending on the breathing frequency of each mouse. Sighs were defined as an increased breath at least twice a normal breath volume. PSAs were defined as apneas that occurred within 20 s of the occurrence of a sigh (Yamauchi et al., 2008). PSAs were further differentiated as (1) sighs followed by <2 apneas, i.e. sigh not followed by apnea, or accompanied by one apnea; and (2) sighs followed by ≥2 apneas, separated at least by one breath.

### CLAMS

CLAMS (Oxymax/CLAMS; Columbus Instruments, Columbus, OH, USA) was used to measure delta oxygen (ΔO_2_) and RER (*V̇*_CO_2__/*V̇*_O_2__) *in vivo*. A previously described protocol was used to conduct these experiments (Koonen et al., 2010). One-month-old mice (*n*=5/genotype) were monitored for 24 h to measure under both light (inactive) and dark (active) cycles for 12 h each.

### Metabolomics – combined direct flow injection and liquid chromatography with tandem mass spectrometry compound identification and quantification

A targeted quantitative metabolomics approach was used to analyze the blood serum samples of Bmp7^ctrl^ and Bmp7^ncko^ mice (*n*=5 mice/genotype) using a combination of direct injection mass spectrometry (AbsoluteIDQ™ kit) with a reverse-phase liquid chromatography with tandem mass spectrometry (LC-MS/MS) kit (BIOCRATES LifeSciences AG, Austria). This kit, in combination with an ABI 4000 Q-Trap (Applied Biosystems/MDS Sciex) mass spectrometer, combines the derivatization and extraction of analytes, and the selective mass-spectrometric detection using multiple reaction monitoring pairs. Isotope-labeled internal standards and other internal standards are integrated in the kit plate filter for metabolite quantification. All the serum samples were analyzed with the AbsoluteIDQ kit using the protocol described in the AbsoluteIDQ user manual. Briefly, serum samples were thawed on ice and vortexed and centrifuged at 13,000 ***g***. Then, 10 µl of each serum sample was loaded onto the center of the filter on the upper 96-well kit plate and dried in a stream of nitrogen. Subsequently, 20 µl of a 5% solution of phenyl-isothiocyanate was added for derivatization. After incubation, the filter spots were dried again using an evaporator. Extraction of the metabolites was then achieved by adding 300 µl of methanol containing 5 mM ammonium acetate. The extracts were obtained by centrifugation into the lower 96-deep well plate, followed by a dilution step with the kit mass spectrometry running solvent. Mass spectrometric analysis was performed on an API4000 Qtrap^®^ tandem mass spectrometry instrument (Applied Biosystems/MDS Analytical Technologies, Foster City, CA, USA) equipped with a solvent delivery system. The samples were delivered to the mass spectrometer by a liquid chromatography method followed by a direct injection method. The Biocrates MetIQ software was used to control the entire assay workflow, from sample registration to automated calculation of metabolite concentrations to the export of data into other data analysis programs. A targeted profiling scheme was used to quantitatively screen for known small-molecule metabolites using multiple reaction monitoring, neutral loss and precursor ion scans. Significantly altered metabolites between Bmp7^ctrl^ and Bmp7^ncko^ mice were identified via a two-tailed unpaired Student's *t*-test.

### Treadmill endurance test

Mice were run on the Exer 3/6 treadmill with a variable speed controller (Columbus Instruments) (Dougherty et al., 2016). For endurance, after initial training, mice were familiarized with a speed of 3 m/min for 2 min, increasing every 2 min by 1 m/min. Distance recording started at a speed of 6 m/min for 6 min, followed by 8 m/min for 10 min, subsequent to which the speed was increased by 2 m/min every 3 min. The time when animals tired was recorded and the distance run calculated. For speed testing, mice were familiarized at 7 m/min for 5 min, and then speed was set to 12 m/min for up to 5 min. Mice were video recorded. For endurance testing, four mice/genotype were recorded. For speed testing, five mice/genotype were recorded.

### Statistics

Results are expressed as means±s.d. For the morphometric µCT, CLAMS and metabolomics analyses, significant differences between Bmp7^ctrl^ and Bmp7^ncko^ mice were estimated by a two-tailed independent unpaired Student's *t*-test, and differences were considered statistically significant when *P*<0.05. Morphometric µCT measurements were conducted by one rater in triplicate. Intra-rater reliability was assessed wherever possible by intra-class correlation (ICC) calculation based on six mice at the 1-month time point, with ICC values greater than 0.9 being considered as excellent reliability (Table S4). For findings from whole-body plethysmography, a statistical comparison of means was performed to compare respiratory (V_T_, fR, *V̇*_E_, number of SAs and sighs, % PSA), metabolic (*V̇*_O_2__, *V̇*_E_/*V̇*_O_2__) and Tb variables between the experimental groups [Bmp7^ctrl^, Bmp7^ncko (r)^ and Bmp7^ncko (a)^ mice] and gas exposure (21% O_2_, 10% O_2_ and 40% O_2_). Two-way repeated measures ANOVA and Holm–Sidak post test were used for each of these comparisons. Comparison of the normoxic T_TOT_, T_i_ and T_e_ among the experimental groups [Bmp7^ctrl^, Bmp7^ncko (r)^ and Bmp7^ncko (a)^ mice] was performed using one-way ANOVA. Values of *P*<0.05 were considered significant.

## Supplementary Material

Supplementary information
