# Supplementary material for: Neural crest-specific loss of Bmp7 leads to midfacial hypoplasia, nasal airway obstruction and disordered breathing, modeling obstructive sleep apnea
Source: Dis Model Mech. 2021 Feb 11;14(2):dmm047738. doi: 10.1242/dmm.047738 (PMC7888714; doi:10.1242/dmm.047738)
Supplement: Supplementary information [file dmm-14-047738-s1.pdf]

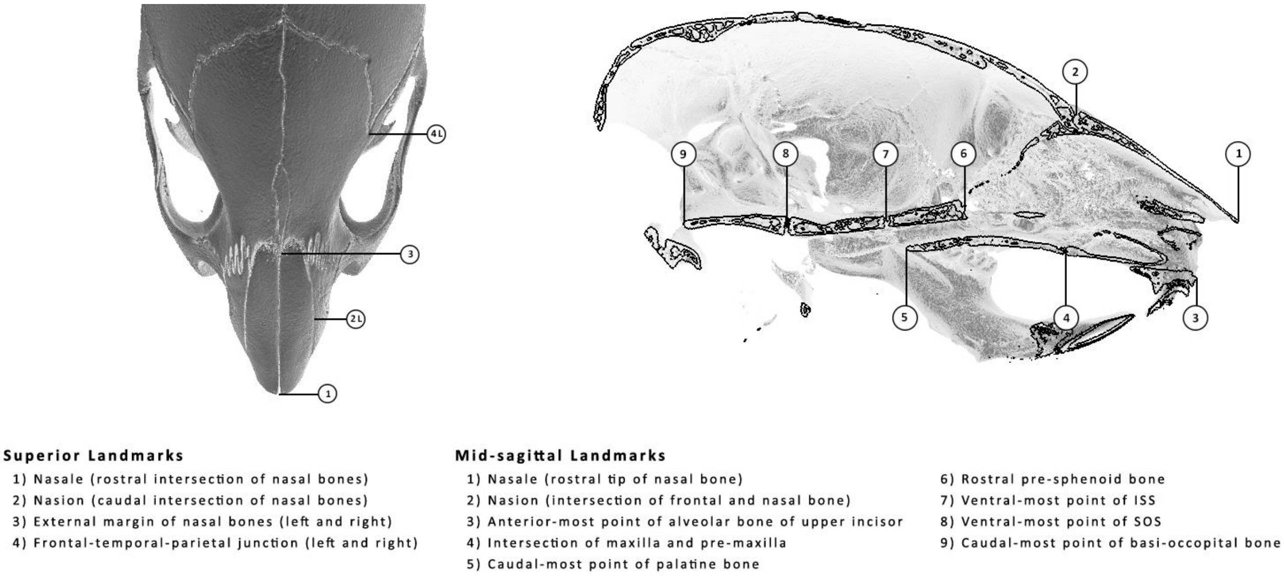

Fig. S1. Diagram of morphometric landmarks and measurements used to characterize craniofacial growth, or lack thereof, in Bmp7^{ctrl} and Bmp7^{ncko} mice. (left panel): superior view of the skull. (right panel): mid-sagittal view of the skull.

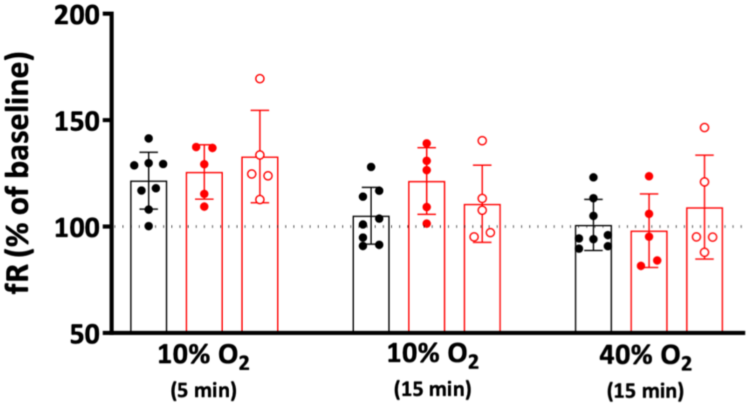

Fig. S2. Respiratory pattern during normoxia in 2-week-old Bmp7^{ctrl} and Bmp7^{ncko} mice. (A) Respiratory frequency (fR). **(B)** Breath cycle duration (TTOT), Inspiratory time (Ti) and Expiratory time (Te). **(C)** Number of apneas/hour. **(D)** Number of sighs/hour. **(E)** Percentage (%) of post sigh apneas (PSA) / total number of sighs with < 2 PSA or ≥ 2 PSA apneas. There was no statistical difference between the groups ($p > 0.05$).

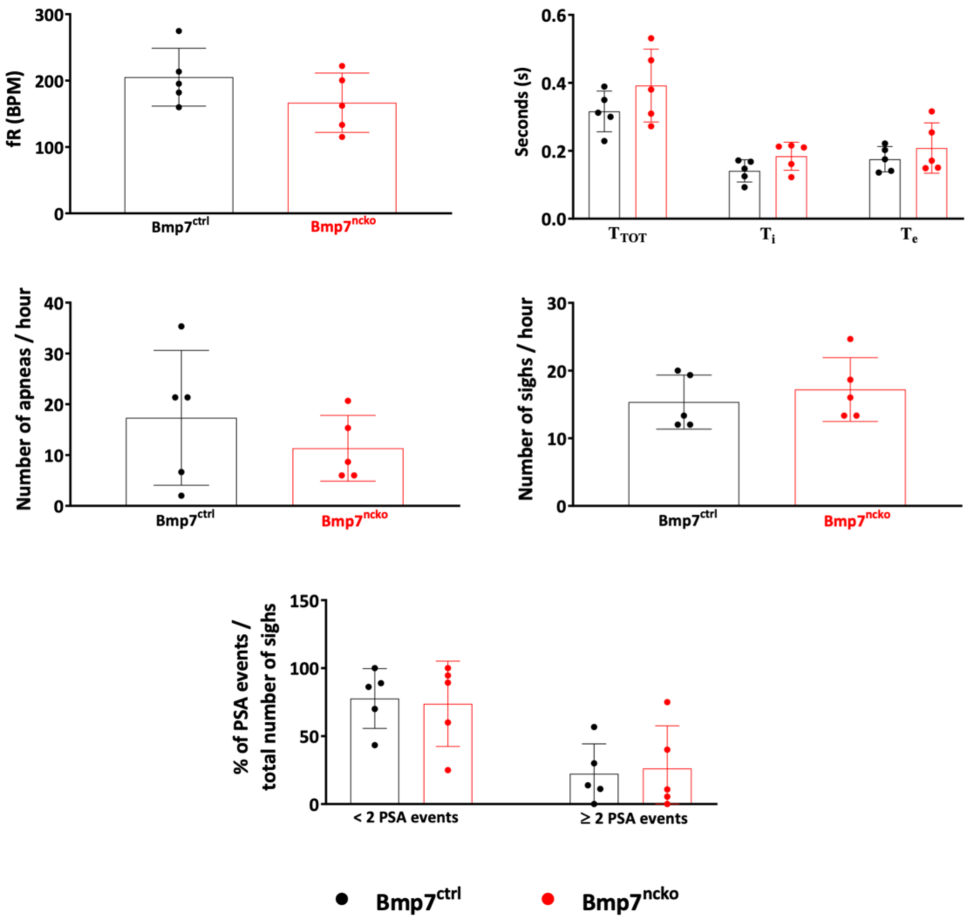

Fig. S3. Respiratory frequency (fR) during hypoxia and hyperoxia exposures. Absolute values from figure 5A shown as percentage (%) of baseline values during 5 and 15 min of hypoxia (10% O2) and 15 min of hyperoxia (40% O2), in $Bmp7^{ctrl}$, $Bmp7^{ncko\ (r)}$ and $Bmp7^{ncko\ (a)}$ mice.

Disease Models & Mechanisms • Supplementary information

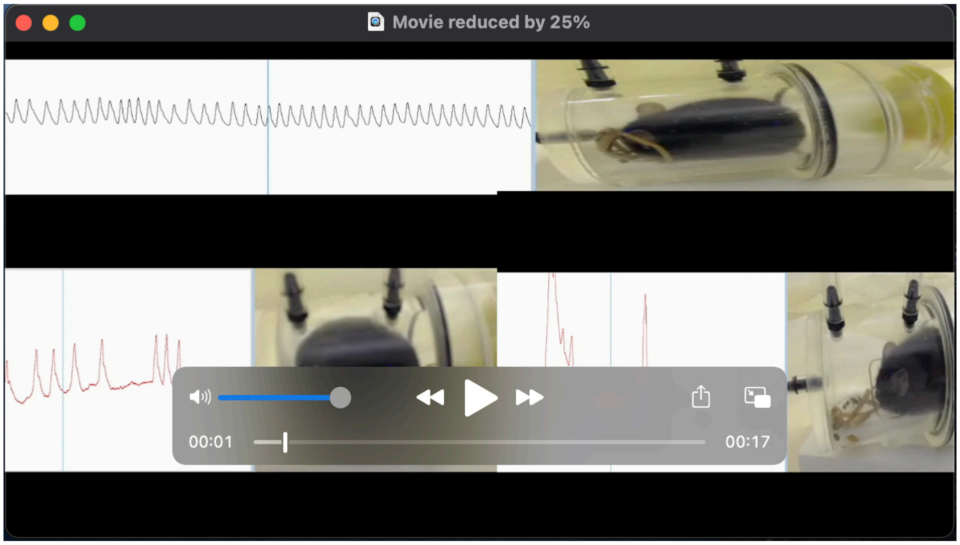

Movie 1. Video representation of Bmp7^{ncko} mice experiencing apneas. Bmp7^{ncko} mice experience spontaneous apnea events Mice were placed in plethysmography chambers and video of the mutant mouse behaviour during baseline measurements was recorded.

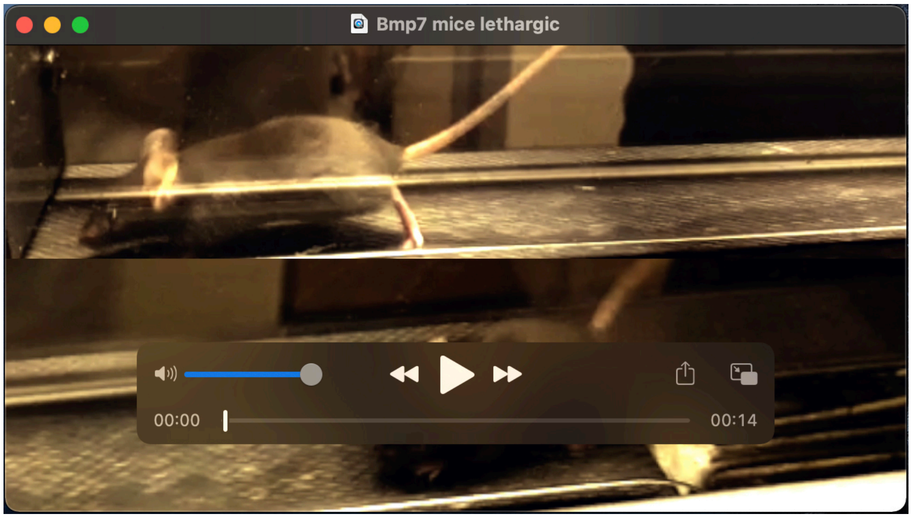

Movie 2. Video representation of Bmp7^{ncko} mice demonstrating lethargy. Bmp7^{ctrl} (top panel) and Bmp7^{ncko} (bottom panel) mice were placed on the treadmill for 5 mins after acclimatization to run. The Bmp7^{ctrl} mice demonstrated no signs of inactivity and rest, however the Bmp7^{ncko} mice demonstrate short period of running followed by long periods of rest.

Table S1. Two-tailed independent t-test statistical comparison of control and mutant morphometric data to accompany Figure 1. P14: 2-week-old mice; P21: 3-week-old mice; P30: 1-month-old mice. n=3 for each age group per genotype.

	Age	Degrees of freedom	T statistic	p-value
Facial length				
	P14	3	1.553238	0.218187
**	P21	4	12.44201	0.00024
*	P30	4	7.073297	0.002108
Snout angle				
	P14	2	-0.51867	0.655675
	P21	2	1.393439	0.298145
	P30	4	-1.83126	0.141021
Frontal bossing				
	P14	3	-1.97315	0.143007
	P21	2	-2.56254	0.12448
	P30	3	2.238238	0.111143
Nasal depression				
	P14	4	0.583792	0.590695
	P21	2	2.566908	0.124132
	P30	3	-0.95634	0.409451
Nasal bone width				
	P14	2	-2.77746	0.108867
	P21	4	0.518786	0.631289
	P30	2	0.243733	0.830158
Nasal bone length				
	P14	4	2.003438	0.115662
	P21	3	2.057673	0.13179
	P30	3	-0.04358	0.96798

Disease Models & Mechanisms • Supplementary information

Table S2. Two-tailed independent t-test statistical results to accompany Figure 2 morphometric data from 1 month old mice. * indicates p<0.05; ** indicates p<0.001. n=3 for each age group per genotype.

	Measurement	Degrees of freedom	T statistic	p-value
*	cranial base angle 1	19	2.607641	0.017301
	cranial base angle 2	23	1.225689	0.232719
	basioccipital length	25	1.669665	0.107462
**	basisphenoid length	25	5.914634	3.58E-06
	presphenoid length	29	0.313573	0.75609
	ethmoid length	29	0.311218	0.757861
**	posterior cranial base length	25	4.27551	0.000244
**	facial length	31	6.013647	1.18E-06
**	snout angle	22	5.541883	1.43E-05
	cranium maxilla angle	23	0.610879	0.54727
**	frontal bossing	25	-5.79575	4.84E-06
**	nasal depression	21	7.335938	3.21E-07
	nasal bone width	22	1.879617	0.07347
*	nasal bone length	31	3.12455	0.003847

Table S3. $Bmp7^{ncko}$ mice showed no changes to cranial base angles and lengths prior to nasal septum deviation. P14: 2-week-old-mice; P21: 3-week-old-mice; n=3 for each age group per genotype.

Measurement	P14 $Bmp7^{ctrl}$	P14 $Bmp7^{ncko}$	p-value	P21 $Bmp7^{ctrl}$	P21 $Bmp7^{ncko}$	p-value
cranial base angle 1	154.93±1.42	151.66±5.99	0.40	150.55±2.71	144.46±6.62	0.13
cranial base angle 2	150.64±2.50	148.18±4.44	0.31	146.48±4.46	143.64±5.20	0.42
basioccipital length	2.89±0.33	2.758±0.15	0.44	3.32±0.71	2.93±0.29	0.29
basisphenoid length	2.712±0.20	2.468±0.19	0.086	2.67±0.31	2.69±0.37	0.92
presphenoid length	2.14±0.12	2.04±0.19	0.35	2.21±0.17	2.14±0.13	0.50
ethmoid length	4.36±0.28	4.5±0.37	0.53	4.95±0.27	4.88±0.22	0.72
posterior cranial base length	8.03±0.26	7.75±0.23	0.11	8.09±0.75	7.67±0.23	0.27

Table S4. Intraclass correlation (ICC) assessment to address intrarater reliability. Three P30 mice of each genotype were landmarked and measured in triplicate by a single rater.

		confidence interval	
mouse ID	ICC	lower	upper
C324	0.999524	0.998854	0.999833
C325	0.999798	0.999513	0.999929
C327	0.999274	0.998253	0.999746
C531	0.996789	0.992222	0.998877
C586	0.997853	0.994838	0.999247
C600	0.998306	0.995721	0.999416
K3308	0.998042	0.995252	0.999315
K3309	0.998773	0.997022	0.999571

Table S5. The variability of tidal volume (V_T), breathing frequency (fR), breath duration (T_{TOT}) and inspiratory time (Ti) (mean ± S.D.) during normoxia in $Bmp7^{ctrl,}$ $Bmp7^{ncko\ (r)}$ and $Bmp7^{ncko\ (a)}$ mice. *P=0.016 for $Bmp7^{ncko\ (r)}$ versus $Bmp7^{ncko\ (a)}$ mice.

	**$Bmp7^{ctrl}$ (n=8)**	**$Bmp7^{ncko\ (r)}$ (n=5)**	**$Bmp7^{ncko\ (a)}$ (n=5)**
**V_T**			
SD1	0.4±0.7	0	0.6±0.8
SD2	1.9±3.4	0	2.7±3.9
**fR**			
SD1	25.1±13.9	18.1±4.0	25.6±7.0
SD2	50.2±17.1	34.5±6.1	50.0±9.0*
**T_{TOT}**			
SD1	0.4±0.7	0	0.6±0.8
SD2	1.1±2.4	0.1	2.8±3.9
**Ti**			
SD1	0.4±0.7	0	0.6±0.8
SD2	1.1±2.4	0	2.7±3.9
